# Optogenetic Stimulation of Vagal Efferent Activity Preserves Left Ventricular Function in Experimental Heart Failure

**DOI:** 10.1016/j.jacbts.2020.06.002

**Published:** 2020-07-15

**Authors:** Asif Machhada, Patrick S. Hosford, Alex Dyson, Gareth L. Ackland, Svetlana Mastitskaya, Alexander V. Gourine

**Affiliations:** a Centre for Cardiovascular and Metabolic Neuroscience, Department of Neuroscience, Physiology and Pharmacology, University College London, London, United Kingdom; b Translational Medicine and Therapeutics, William Harvey Research Institute, Queen Mary University of London, London, United Kingdom; c Clinical Physiology, Division of Medicine, University College London, London, United Kingdom

**Keywords:** autonomic nervous system, heart failure, myocardial infarction, neuromodulation, vagus nerve stimulation, ABP, arterial blood pressure, DVMN, dorsal motor nucleus of the vagus nerve, eGFP, enhanced green fluorescent protein, GRK2, G-protein−coupled receptor kinase 2, LAD, left anterior descending coronary artery, LV, left ventricle, LV dP/dt_MAX_, maximum rate of rise of left ventricular pressure, LVEDP, left ventricular end-diastolic pressure, LVESP, left ventricular end-systolic pressure, LVP, left ventricular pressure, LVV, lentiviral vector, MI, myocardial infarction, VNS, vagus nerve stimulation

## Abstract

Large clinical trials designed to test the efficacy of vagus nerve stimulation (VNS) in patients with heart failure did not demonstrate benefits with respect to the primary endpoints. The nonselective nature of VNS may account for the failure to translate promising results of preclinical and earlier clinical studies. This study showed that optogenetic stimulation of vagal pre-ganglionic neurons transduced to express light-sensitive channels preserved left ventricular function and exercise capacity in a rat model of myocardial infarction−induced heart failure. These data suggested that stimulation of vagal efferent activity is critically important to deliver the therapeutic benefit of VNS in heart failure.

## Highlights

• This study was designed to determine the effect of selective optogenetic simulation of vagal efferent activity on left ventricular function in an animal (rat) model of MI-induced heart failure.
• Optogenetic stimulation of dorsal brainstem vagal pre-ganglionic neurons transduced to express light-sensitive channels preserved LV function and exercise capacity in animals with MI.
• The data suggest that activation of vagal efferents is critically important to deliver the therapeutic benefit of VNS in chronic heart failure.

## Visual Abstract

**Figure:**
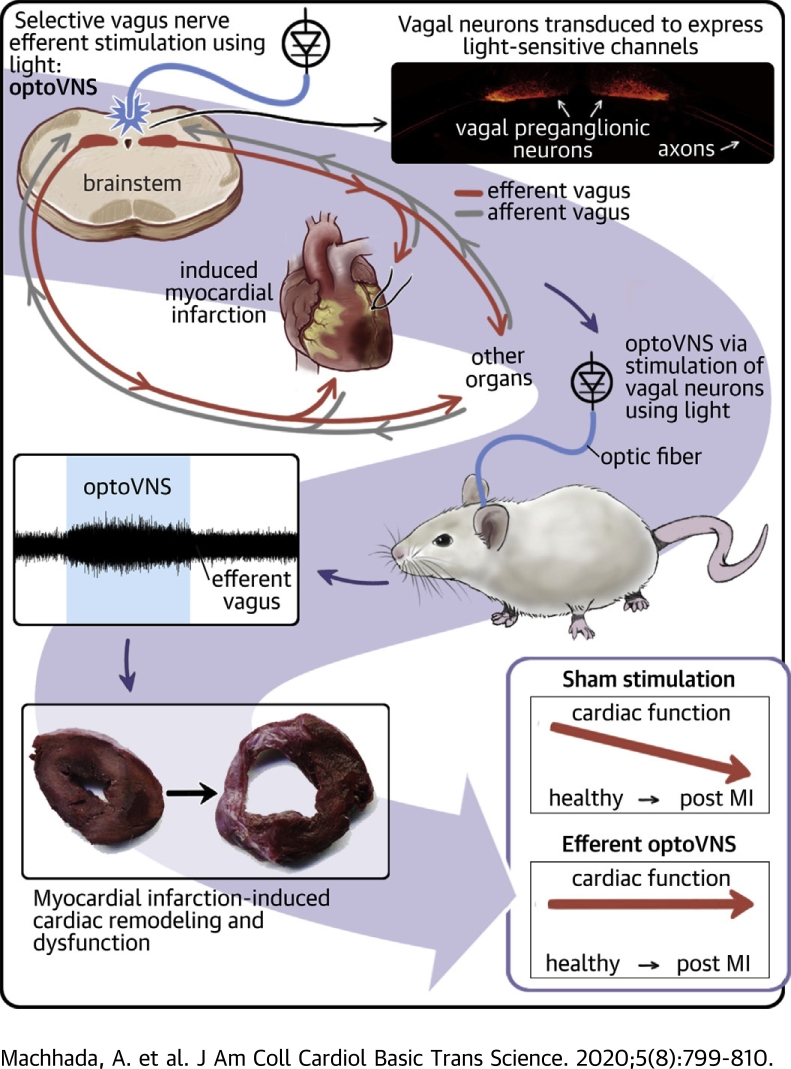

Chronic heart failure is 1 of the most common causes of morbidity and mortality in the developed world. Established pharmacological treatment of heart failure that involves several drug classes, including β-blockers and inhibitors of renin-angiotensin-aldosterone system, improves symptoms and reduces mortality. Yet, drug therapy remains insufficient because cardiac function continues to deteriorate over time, and most patients have poor prognosis (1).

Autonomic dysfunction characterized by sympathetic activation and parasympathetic (vagal) withdrawal accelerates the development and progression of the disease (2, 3, 4). The current gold standard pharmacological treatment includes drugs that limit the sympathetic effects on the heart and kidneys (e.g., β-blockers). However, stimulation of vagal efferent activity to increase parasympathetic tone and redress autonomic balance as a treatment for heart failure has proved to be more difficult to achieve (5,6). Patients with heart failure on optimal medical therapy with persistent autonomic dysfunction have the worst prognosis (7).

Electrical vagus nerve stimulation (VNS) has been shown to reduce the extent of an acute myocardial infarction (MI) (8, 9, 10, 11) and slow the progression of myocardial remodeling, as well as atrial and ventricular dysfunction in animal models of chronic heart failure (12, 13, 14, 15, 16, 17). In the first human trial that involved 32 patients with heart failure, De Ferrari et al. (18) demonstrated that electrical stimulation of the right vagus nerve using implanted devices improved New York Heart Association functional class, left ventricular (LV) function, and quality of life. However, subsequent multicenter trials designed to test VNS efficacy in large cohorts of patients with heart failure reported mixed results. Premchand et al. (19) showed improvements of the ejection fraction and symptoms in another noncontrolled study that involved 60 patients (ANTHEM-HF [Autonomic Neural Regulation Therapy to Enhance Myocardial Function in Heart Failure] study), whereas Zannad et al. (20) and Gold et al. (21) reported no effect of VNS on LV remodeling and function or mortality in trials that involved 86 (NECTAR-HF [Neural Cardiac Therapy for Heart Failure]) and 707 (INOVATE-HF [Increase of Vagal Tone in Chronic Heart Failure]) patients. However, there were reported improvements in quality of life (20).

The mechanisms underlying the potential benefit of VNS in heart failure remain poorly understood. The cervical vagus is a mixed nerve containing sensory and motor fibers at an approximate ratio of 4:1. The nerve bundle contains branches that innervate most of the viscera and sparse sympathetic fibers. There is no experimental data to suggest that the VNS beneficial effects on the failing heart observed in animal and some human studies are due to the recruitment of afferent (sensory) or efferent (motor) vagal fibers (or both) by the electrical current pulses applied to the nerve at the cervical level. Although attempts had been made to design VNS devices for preferential stimulation of vagal efferents (5,18), electrical properties of the nerve fibers that constitute the human vagus nerve make selective fiber recruitment virtually impossible to achieve.

Further refinement of VNS as a potential treatment for human heart failure may require development of alternative methods of selective stimulation of specific groups or subsets of vagal fibers. Selective stimulation would be expected to maximize the efficacy and limit the side effects associated with the recruitment of fibers that may not necessarily confer any benefit on the failing heart (22). The optogenetic approach involving transduction of mammalian neurons to express the native or modified light-sensitive channels from algae and activation of these neurons using light provides the required level of selectivity for experimental studies (23). Recently, we and other laboratories used optogenetic techniques to study the nervous mechanisms controlling the heart (11,24,25). Our research group demonstrated that stimulation of approximately 300 to 400 vagal pre-ganglionic neurons in the dorsal motor nucleus of the vagus nerve (DVMN) markedly reduced the extent of MI in an animal model (11). These data provided direct evidence of effective cardioprotection by selective stimulation of vagal efferent activity. In the present study, we genetically targeted the DVMN neurons to express the light-sensitive channel ChiEF and determined the effect of selective stimulation of the DVMN vagal efferent projections using light (optoVNS) on LV function in a rat model of MI-induced heart failure.

## Methods

All the experiments were performed in accordance with the European Commission Directive 2010/63/EU (European Convention for the Protection of Vertebrate Animals used for Experimental and Other Scientific Purposes) and the United Kingdom Home Office (Scientific Procedures) Act (1986) with project approval from the University College London Institutional Animal Care and Use Committee. The rats were group housed and maintained on a 12-h light cycle (lights on 0700) and had ad libitum access to water and food.

### Genetic targeting of the DVMN vagal pre-ganglionic neurons

Vagal pre-ganglionic neurons of the DVMN express transcriptional factor Phox2 and were targeted to express a light-sensitive chimeric channelrhodopsin derivative ChIEF fused with a fluorescent protein tdTomato (ChIEFtdTomato) or enhanced green fluorescent protein (eGFP) (control transgene) using lentiviral vectors (LVV). Transgene expression was driven under the control of a Phox2-activated promoter PRSx8 (26). Validation of the specificity of vectors in transducing the DVMN neurons was described in detail previously (11,27). Pulses of blue (445 nm) light trigger precisely timed depolarizations and action potential firing of the DVMN neurons transduced to express ChIEF (11).

**Figure 1:**
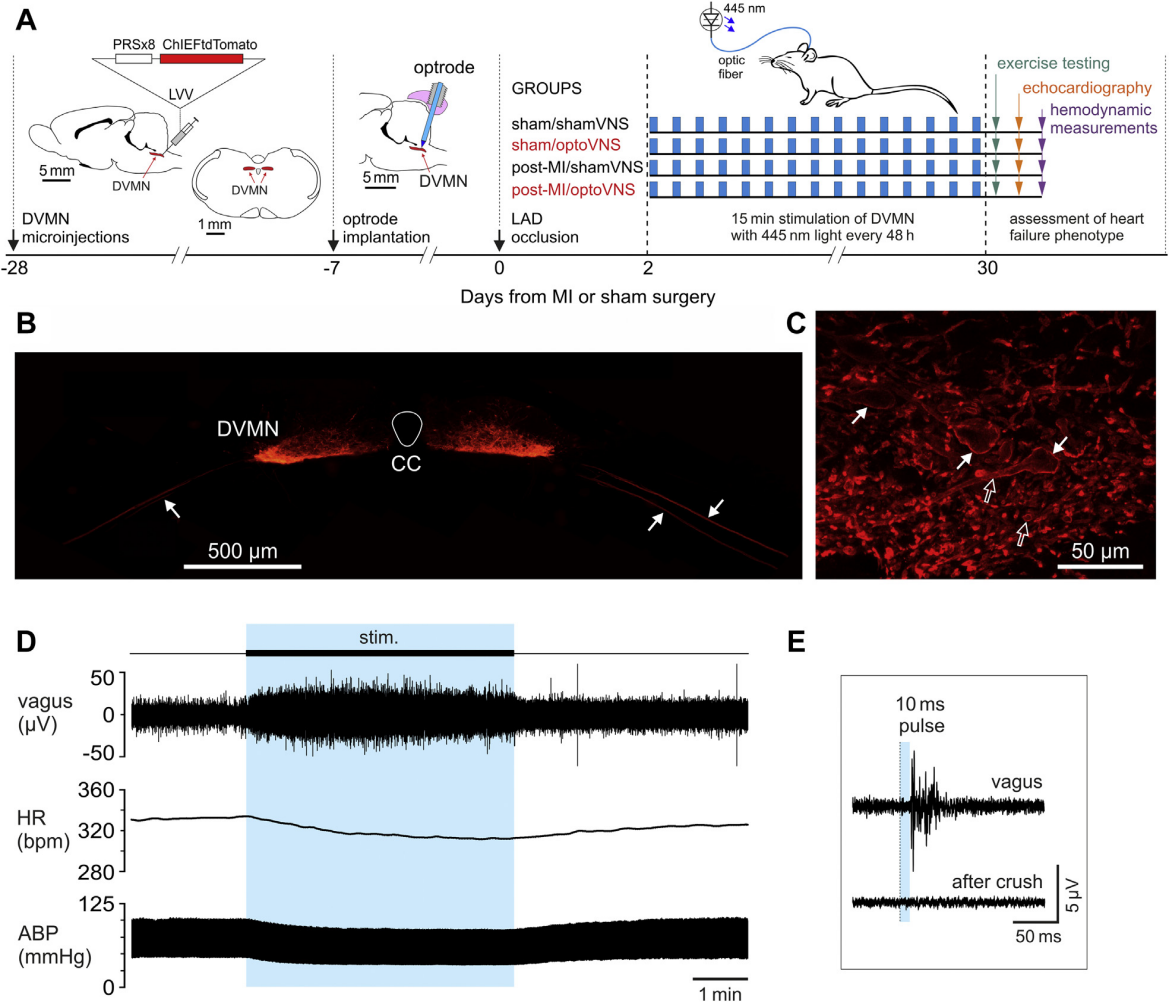
Design of the Study and Genetic Targeting of Vagal Pre-Ganglionic Neurons to Express Light-Sensitive Channel ChIEF for Selective Stimulation of Vagal Efferent Activity Using Light **(A)** The experimental timeline, study protocols, and schematic drawings of the key methods. The rat brain is drawn in sagittal and coronal projections to illustrate the anatomical position of the dorsal motor nucleus of the vagus nerve (DVMN) targeted with a lentiviral vector (LVV) to express ChIEFtdTomato under the control of the PRSx8 promoter. The caudal aspect of the rat brain is also drawn in the sagittal projection to illustrate the approximate positioning of the optrode implant to stimulate the cell bodies of DVMN vagal pre-ganglionic neurons using light. **(B)** A photomicrograph of a representative coronal section of the rat dorsal brainstem taken at low magnification illustrating a representative example of ChIEFtdTomato expression in the caudal region of the DVMN. **Arrows** point at ventrally projecting axons of the transduced DVMN neurons, forming the efferent vagus nerve. **(C)** A high magnification image illustrating ChIEFtdTomato expression by vagal preganglionic neurons of the DVMN. **White arrows** point at neuronal cell bodies; **open arrows** point at severed neuronal axons displaying specific membrane localization of ChIEFtdTomato. **(D)** Representative raw data illustrating the effect of light stimulation (445 nm, 10 ms pulse, 15 Hz) of DVMN neurons expressing ChIEFtdTomato on heart rate (HR), arterial blood pressure (ABP), and the efferent activity of the cervical vagus nerve in a urethane-anesthetized rat. **(E)** Evoked efferent vagus nerve action potential firing induced by 10 ms pulse of light delivered at 1 Hz to stimulate the DVMN vagal preganglionic neurons expressing ChIEFtdTomato. bpm = beats/min; CC = central canal; LAD = left anterior descending coronary artery; MI = myocardial infarction.

Male Sprague-Dawley rats (150 to 200 g; n = 68) were anesthetized with a combination of ketamine (60 mg/kg intramuscularly) and medetomidine (250 μg/kg intramuscularly). Adequate depth of surgical anesthesia was maintained and confirmed by the absence of a withdrawal response to a paw pinch. With the head of the animal in a stereotaxic frame, a midline dorsal neck incision was made to expose the dorsal brainstem surface. Caudal populations of the DVMN neurons were targeted bilaterally with 1 microinjection on each side that delivered viral particles of either LVV-PRSx8-eGFP or LVV-PRSx8-ChIEFtdTomato (Figure 1A). The microinjections (0.5 μl at a rate of 0.1 μl/min) were made at 0.5 mm rostral, 0.6 mm lateral, and 0.8 mm ventral from the calamus scriptorius. After the microinjections, the wound was sutured, and anesthesia was reversed with atipamezole (1 mg/kg; intramuscularly). Post-operatively, the animals received buprenorphine (0.05 mg/kg/day, subcutaneously) analgesia for 3 days. No complications were observed after the surgery, and the animals gained weight normally.

### Optrode implantation

Two weeks after the microinjections of viral vectors, the animals were anesthetized with ketamine (60 mg/kg intramuscularly) and medetomidine (250 μg/kg intramuscularly), and an optrode (Art Photonics, Berlin, Germany) was implanted to reach the dorsal surface of the brainstem for optimal light delivery to the groups of transduced vagal neurons. Two screws were placed into the skull, and the implant was secured in place with dental acrylic (Figure 1A). Anesthesia was reversed with atipamezole (1 mg/kg intramuscularly). Carprofen (5 mg/kg/day subcutaneously) was given for post-operative analgesia for 3 days, and the animals were allowed to recover for 7 days. Rats were 300 to 350 g at the time of the main experiment.

### Recording the efferent activity of the vagus nerve

Rats (n = 4) transduced to express ChiEFtdTomato by the DVMN neurons were anesthetized with urethane (induction: 1.3 g/kg, intraperitoneally; maintenance: 10 to 25 mg/kg/h intravenously) and instrumented as described in detail previously (28,29). Adequate anesthesia was ensured by maintaining stable levels of arterial blood pressure (ABP) and heart rate and showing lack of responses to a paw pinch. The femoral artery and vein were cannulated for measurements of blood pressure and administration of anesthetic, respectively. The trachea was cannulated, and the animal was mechanically ventilated with air supplemented with oxygen using a positive pressure ventilator with a tidal volume of approximately 1 ml/100 g of body weight and ventilator frequency similar to the resting respiratory rate (∼60 strokes/min). Partial pressure of oxygen, partial pressure of carbon dioxide, and pH of arterial blood were measured regularly and kept within physiological ranges (partial pressure of oxygen: 95 to 105 mm Hg; partial pressure of carbon dioxide: 35 to 45 mm Hg, and pH 7.35 to 7.45) by adjusting the tidal volume and/or ventilator frequency as well as the level of supplemental oxygen. Body temperature was maintained at 37.0 ± 0.5°C. The right vagus nerve was dissected, isolated from the surrounding tissues, placed on silver wire recording electrodes, and covered with dental impression material. The recorded signal was amplified (×10,000), filtered (80 to 1,500 Hz), and sampled at a rate of 5 kHz using a Power1401 interface and Spike2 software (Cambridge Electronic Design, Cambridge, United Kingdom).

### Model of MI-induced heart failure

MI leading to progressive impairment of LV function in rats was induced using a left anterior descending coronary artery (LAD) occlusion technique described in detail in the published reports (30). One week following optrode implantation (Figure 1A), the rats were anesthetized with ketamine (60 mg/kg intramuscularly) and medetomidine (250 μg/kg intramuscularly), and artificially ventilated following endotracheal intubation. A left thoracotomy was performed to expose the heart, the pericardium was opened, the heart was exteriorized, and the LAD was ligated just below the left atrial appendage using 4-0 braided silk suture. Successful coronary occlusion was confirmed by pallor of the anterior LV wall. Sham ligations involved the same sequence of procedures, including placement of the suture through the ventricular wall but without the occlusive tying. The chest incision was closed, the lungs were re-inflated, and artificial ventilation was discontinued once the animal started to breathe spontaneously. Anesthesia was reversed with atipamezole (1 mg/kg intramuscularly). Carprofen (5 mg/kg/day subcutaneously for 3 days) was given for post-operative analgesia. Post-operative mortality within the first 48 h after MI was 26%. All animals that survived beyond 48 h post-MI also survived the remainder of the study period.

### Experimental groups

On the basis that the minimum physiologically relevant effect of efferent vagus stimulation would be to prevent reduction in the ejection fraction after LAD occlusion by 8%, we estimated that at least 8 animals in each of the 4 experimental groups would be required (α = 0.01; 1-β = 0.9). The rats were randomized into 4 experimental groups (the rationale for the number of animals included in each of the groups at the beginning of the study is given in the following): 1) sham/shamVNS (n = 8): sham-operated animals expressing eGFP in the DVMN; 2) sham/optoVNS (n = 10): sham-operated animals expressing ChiEFtdTomato in the DVMN; 3) post-MI/shamVNS (n = 13): animals with LAD occlusion expressing eGFP in the DVMN; and 4) post-MI/optoVNS (n = 19): animals with LAD occlusion expressing ChiEFtdTomato in the DVMN (Figure 1A). Ten animals were included in sham/optoVNS group to allow for 20% dropout rate due to a potential lack of (or weak) ChiEFtdTomato expression in the DVMN due to variations of titer between different preparations of the vector. Thirteen animals were included in post-MI/shamVNS group to allow for an approximate 40% dropout of subjects that developed small infarcts (<30%). Nineteen rats were included in post-MI/optoVNS to allow for a potential 20% dropout rate due to lack of ChiEFtdTomato expression and/or 40% dropout due to the development of small infarcts.

The dorsal brainstem was illuminated in all the animals via the implanted optrode with 445-nm light pulses (10 ms, 15 Hz) for 15 min every 48 h for 4 weeks, which commenced 48 h after the LAD occlusion or sham surgery (Figure 1A). To minimize the stress of the animals, light stimulation was performed under mild sedation (1% isoflurane in 1:1 oxygen/air mixture).

### Evaluation of exercise capacity

The exercise capacity of experimental rats was assessed using a single lane treadmill (Panlab Harvard Apparatus, Barcelona, Spain) as previously described (24,31). To determine the exercise capacity, treadmill speed was raised in increments of 5 cm/s every 5 min after an initial 15 min at 20 cm/s until the animal was unable to maintain pace with the moving belt and entered the stationary platform at the end of the treadmill 4 times within a 2-min period, which was the humanely defined point of exhaustion. The distance covered by the animal was recorded, and exercise capacity expressed as work done in Joules.

### Echocardiography

The animals were anesthetized with isoflurane (induction 4%, maintenance 2% in 1:1 oxygen/air mixture). Echocardiographic assessment of LV function was performed using a Vevo 2100 high-resolution ultrasound system with a MS250 13-24MHz linear array transducer (VisualSonics, Toronto, Ontario, Canada), with simultaneous heart rate monitoring (lead II electrocardiography). In a B-mode acquisition, the ejection fraction was determined using a parasternal long-axis view of the LV, measuring the length of the ventricle at the end of systole and diastole. Three perpendicular short-axis images were acquired equidistantly along the length of the LV to segment the endocardial border. The LV volumes were approximated using Simpson’s rule (32). Using an apical 4-chamber view in Doppler mode, blood flow velocity across the mitral valve was also measured. The ratio of flow velocities during the early and atrial ventricular filling phases (E/A ratio) was calculated to assess diastolic function (33).

### Hemodynamic studies

After the echocardiographic study, all animals underwent invasive hemodynamic assessment under urethane (1.3 g/kg intraperitoneally) anesthesia. Adequate depth of anesthesia was ensured by stable levels of ABP and heart rate in addition to the absence of a withdrawal response to a paw pinch. The femoral artery was cannulated for the purpose of measuring ABP. The trachea was cannulated, and the animal was artificially ventilated with room air using a positive pressure ventilator (Harvard Apparatus, Holliston, Massachusetts) with a tidal volume of approximately 2.5 ml and a frequency similar to the spontaneous breathing rate (∼60 strokes/min). Body temperature was maintained at 37.0 ± 0.5°C. A 2-F Millar pressure catheter (model SPR-407; Millar Instruments, Houston, Texas) was advanced via the right carotid artery into the LV chamber to record LV pressure (LVP). Following 30 min of stabilization, LVP, ABP, and standard lead II electrocardiogram were recorded for 15 min using a Power1401 interface and Spike2 software (Cambridge Electronic Design, Cambridge, United Kingdom). The ABP averaged waveforms were used to calculate the mean arterial pressure. The LVP average waveforms were used to calculate LV end-systolic pressure (LVESP) and LV end-diastolic pressure (LVEDP). The first differential of LVP, LV dP/dt was calculated to determine its maximum, LV dP/dt_MAX_. At the end of the hemodynamic studies, the heart was arrested with potassium chloride and removed for the assessment of infarct size.

### Assessment of infarct size

The extent of MI was determined as described in detail previously (30,34,35). Briefly, the LV was cut into 5 to 6 transverse slices of approximately 1.5 mm thickness. The images of the slices were taken, and the length of the entire endocardial and epicardial circumferences, as well as the length of the scar muscle for endocardial and epicardial surfaces, were determined by computerized planimetry using ImageJ software (National Institutes of Health, Bethesda, Maryland). The ratio of the length of the scar and the entire surface circumferences defined the infarct size for endocardial and epicardial surfaces. Infarct size (percentage) was calculated as an average of the infarcted endocardial and epicardial surfaces. The extent of myocardial necrosis of these well-healed infarcts was determined from the circumference rather than from the volume of the infarct because the latter underestimates the initial loss of the myocardium (30).

### Histology

At the end of the hemodynamic study, the brains were removed and fixed in 4% phosphate-buffered (0.1 M, pH 7.4) paraformaldehyde solution. After cryoprotection (30% sucrose), the brainstem was isolated, and a sequence of transverse slices (30 μm) was cut to determine the extent of ChiEFtdTomato or eGFP expression by the DVMN neurons.

### Data analysis

Recordings of the vagus nerve activity and cardiovascular variables were analyzed using Spike2 software. Differences between the experimental groups were assessed using GraphPad Prism 6 software (GraphPad, San Diego, California). Comparisons were made using 2-way analysis of variance followed by Sidak’s p value correction for multiple comparisons or Student’s *t-*test, as appropriate. Data were reported as individual values and mean ± SEM. Differences with p < 0.05 were considered significant.

## Results

Expression of ChIEFtdTomato by the vagal pre-ganglionic neurons residing in the caudal regions of the left and right DVMN was confirmed in all the animals that received bilateral microinjections of LVV-PRSx8-ChIEFtdTomato (Figures 1B and 1C). Strong tdTomato fluorescence was observed in the ventral projections of the transduced DVMN neurons, forming the efferent vagus nerve (Figure 1B). Pulses of 445 nm light (10 ms) applied to the dorsal brainstem of rats transduced to express ChIEFtdTomato by the DVMN vagal pre-ganglionic neurons triggered a volley of action potentials in the cervical vagus with a mean delay of 27 ± 2 ms (n = 4) (Figures 1D and 1E). Calculated conduction velocity of 0.8 m/s was consistent with the properties of the rat DVMN cardiac vagal pre-ganglionic neurons that have C-fiber axons (36). Stimulation of DVMN neurons expressing ChIEF with 10-ms pulses applied at a frequency of 15 Hz led to a robust increase in vagal efferent activity and was associated with a reduction in heart rate (by 30 ± 12 beats/min; 8.5% reduction; p = 0.037) and mean ABP (by 15 ± 4 mm Hg) during the period of stimulation (Figure 1D).

**Figure 2:**
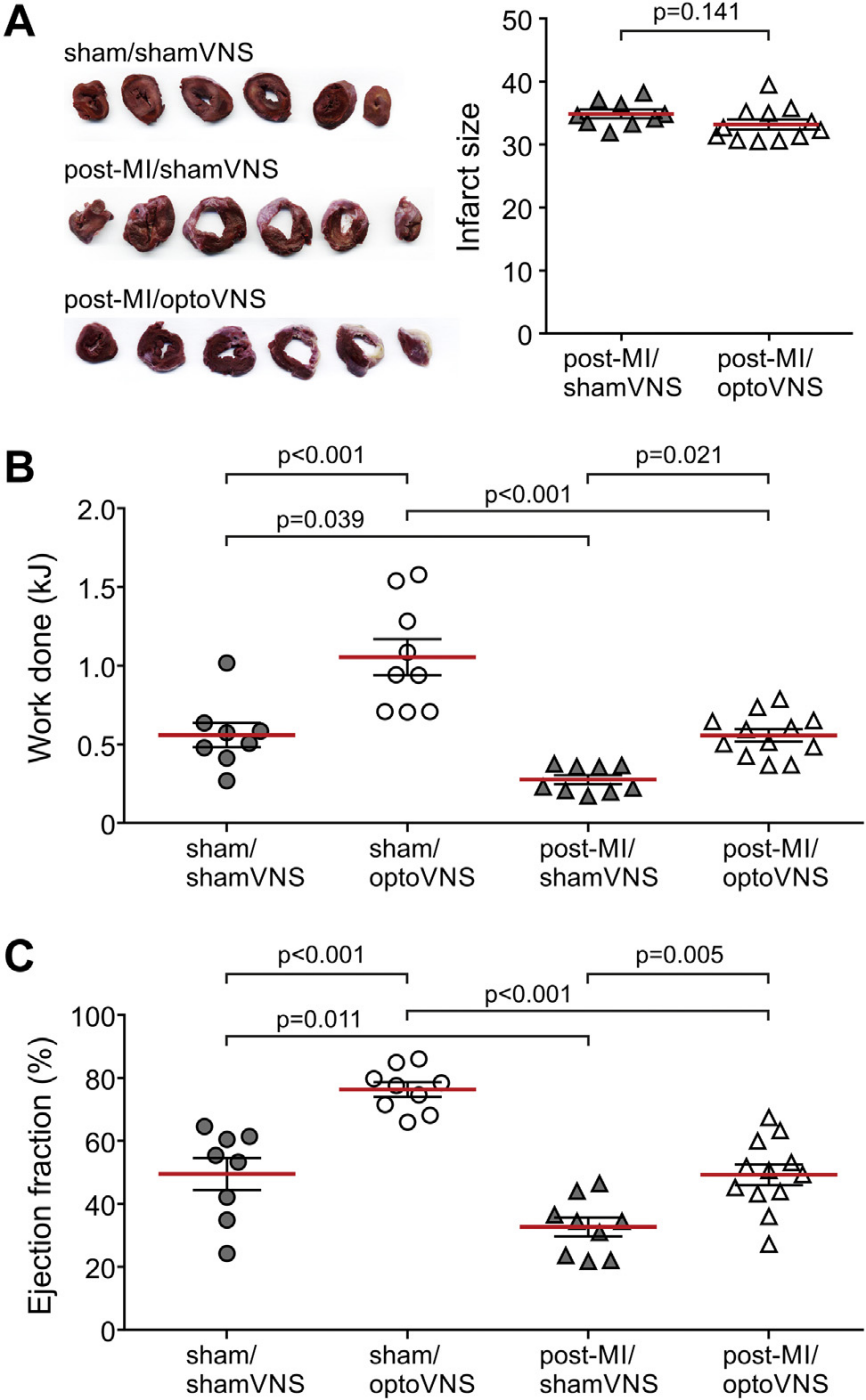
Optogenetic Stimulation of Vagal Efferent Activity Improves Exercise Capacity and Ejection Fraction in MI-Induced Heart Failure in Rats **(A)** Representative sections of the left ventricle (LV) from a sham-operated animal (sham/sham vagus nerve stimulation [VNS] group), an animal with MI expressing enhanced green fluorescent protein (eGFP) in the DVMN after 4 weeks of sham-stimulation (post-MI/shamVNS group), and an animal with MI expressing ChiEFtdTomato in the DVMN after 4 weeks of efferent vagus stimulation using light (post-MI/optoVNS group). Summary data illustrate infarct sizes determined by computerized planimetry of LV sections of rats transduced to express eGFP or ChiEFtdTomato by the DVMN neurons after 4 weeks of light stimulation commencing 2 days after the occlusion of the left anterior descending artery (Student’s *t-*test). **(B and C)** Summary data illustrating the exercise capacity and LV ejection fraction of rats transduced to express eGFP or ChiEFtdTomato by the DVMN neurons after 4 weeks of light stimulation commencing 2 days after the occlusion of the left anterior descending artery or sham surgery. Data are presented as individual values and mean ± SEM. Comparisons are made using 2-way analysis of variance followed by Sidak’s correction for multiple comparisons. Abbreviations as in Figure 1.

The average infarct size in the post-MI/shamVNS group was 33.4 ± 1.6 (n = 13); in the post-MI/optoVNS group it was 29.9 ± 1.3 (n = 19; p = 0.09). Four animals in the post-MI/shamVNS group and 7 animals in the post-MI/optoVNS experimental group developed small infarcts (<30%). In accord with the study design, the data obtained in 9 animals in the post-MI/shamVNS group and 12 animals in the post-MI/optoVNS group that developed infarcts ≥30% were included in the analysis. There was no difference in mean infarct size between the 2 groups (p = 0.14) (Figure 2A).

Exercise capacity was significantly lower in post-MI rats expressing eGFP in the DVMN compared with sham-operated animals (0.28 ± 0.03 kJ in post-MI/shamVNS group vs. 0.56 ± 0.08 kJ in sham/shamVNS group; p = 0.039) (Figure 2B). Optogenetic stimulation of the DVMN neurons expressing ChIEFtdTomato enhanced the exercise capacity in sham-operated animals (1.05 ± 0.12 kJ in sham-operated rats expressing ChiEFtdTomato vs. 0.56 ± 0.08 kJ in sham-operated rats expressing eGFP; p < 0.001) (Figure 2B) and improved the exercise capacity in animals with LAD occlusion (0.56 ± 0.04 kJ in post-MI rats expressing ChiEFtdTomato vs. 0.28 ± 0.03 kJ in post-MI rats expressing eGFP; p = 0.021) (Figure 2B).

Development of MI-induced heart failure in this model was evident from a significant reduction of ejection fraction (32.7 ± 3.0% in the post-MI/shamVNS group vs. 49.6 ± 5.1% in the sham/shamVNS group; p = 0.011) (Figure 2C), which indicated systolic dysfunction. Optogenetic stimulation of vagal pre-ganglionic neurons prevented MI-induced reduction in the ejection fraction (49.3 ± 3.3% in post-MI rats expressing ChiEFtdTomato vs. 32.7 ± 3.0% in post-MI rats expressing eGFP; p = 0.005). Significant increases in contractility were observed in sham-operated animals. Optogenetic stimulation of the DVMN increased the ejection fraction by 26% (76.3 ± 2.3% in sham-operated rats expressing ChiEFtdTomato vs. 49.6 ± 5.1% in sham-operated rats expressing eGFP; p < 0.001). There were no differences in E/A ratio and heart rate (measured under isoflurane anesthesia) between the experimental groups (Table 1).

**Table 1:** Hemodynamic and Other Group Data

	Sham MI		Post-MI	
	shamVNS (n = 8)	optoVNS (n = 9)	shamVNS (n = 9)	optoVNS (n = 12)
Body weight (g)	431 ± 18	406 ± 29	453 ± 18	391 ± 5
Infarct size	—	—	34.9 ± 0.7	33.2 ± 0.8
HR (beats/min, under isoflurane)	348 ± 9	380 ± 12	364 ± 12	389 ± 12
Ejection fraction (%)	49.6 ± 5.1	76.3 ± 2.3	32.7 ± 3.0	49.3 ± 3.3
E/A ratio	1.2 ± 0.1	1.6 ± 0.1	0.9 ± 0.1	1.2 ± 0.1
HR (beats/min, under urethane)	371 ± 15	380 ± 19	363 ± 19	380 ± 13
dP/dt_min_ (mm Hg/s)	−6,136 ± 269	−6,774 ± 391	−4,348 ± 275	−5,550 ± 176
Pulse pressure (mm Hg)	52 ± 4	51 ± 2	36 ± 4	51 ± 5
LV to body weight ratio	2.12 ± 0.07	1.92 ± 0.06	2.19 ± 0.08	2.24 ± 0.05
RV to body weight ratio	0.38 ± 0.01	0.37 ± 0.01	0.42 ± 0.02	0.36 ± 0.02
Lung fluid (%)	77.5 ± 0.3	77.8 ± 0.5	77.3 ± 0.3	78.4 ± 0.3

Values are mean ± SEM.

dP/dt_min_ = maximum rate of left ventricular pressure decrease; HR = heart rate; LV = left ventricle; MI = myocardial infarction; RV = right ventricle; VNS = vagus nerve stimulation.

**Figure 3:**
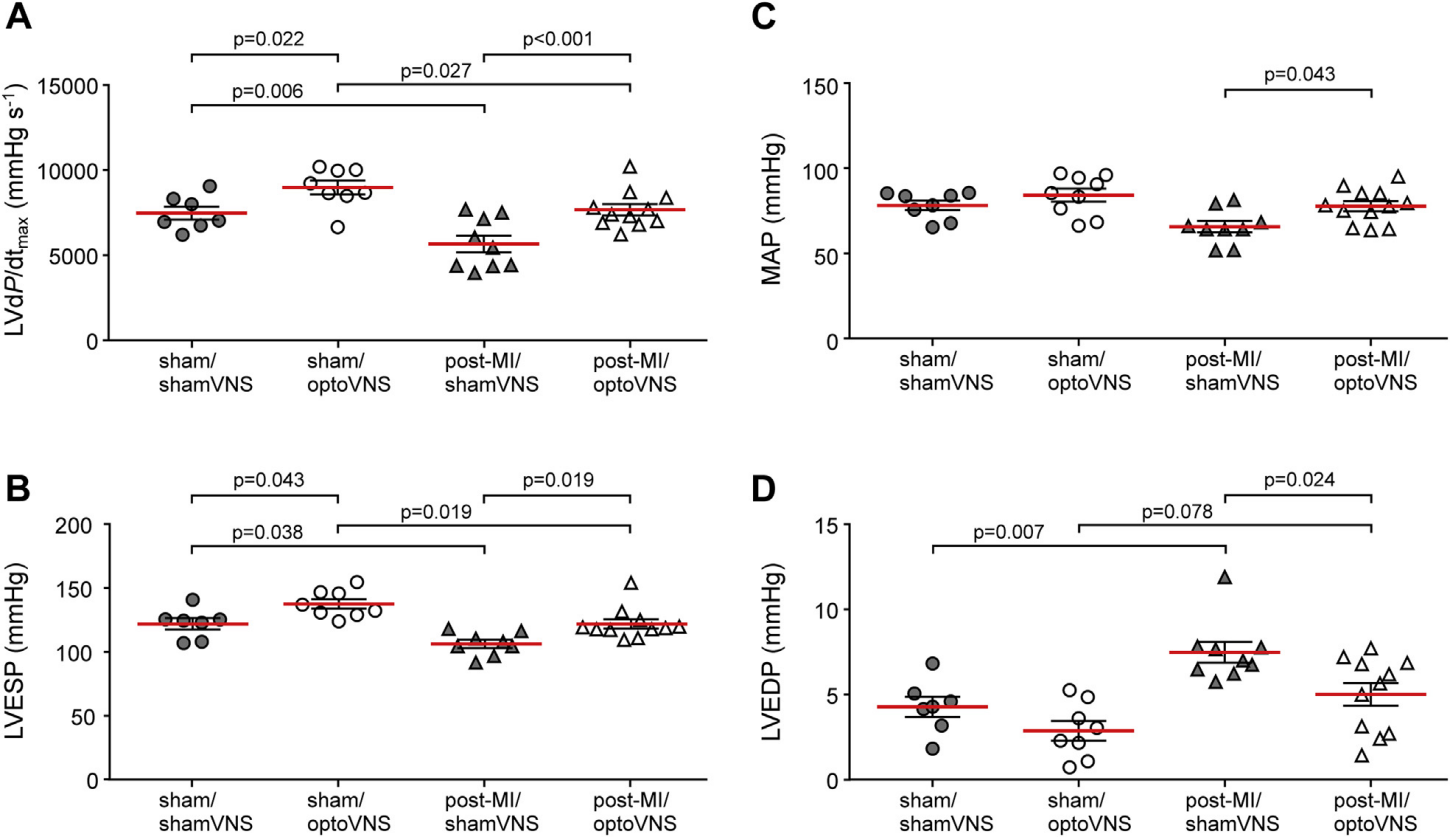
Optogenetic Stimulation of Vagal Efferent Activity Preserves LV Function in MI -Induced Heart Failure in Rats Summary data illustrating the values of **(A)** the maximum first differential of LV pressure (LV dP/dt_max_), **(B)** LV end-systolic pressure (LVESP), **(C)** mean arterial blood pressure (MAP), and **(D)** LV end-diastolic pressure (LVEDP) in rats transduced to express eGFP or ChiEFtdTomato by the DVMN neurons after 4 weeks of light stimulation commencing 2 days after the occlusion of the left anterior descending artery or sham surgery. Data are presented as individual values and mean ± SEM. Comparisons are made using 2-way analysis of variance followed by Sidak’s correction for multiple comparisons. Abbreviations as in Figures 1 and 2.

Matching improvements in LV function as a result of optogenetic stimulation of the DVMN neuronal activity were recorded during invasive assessment of cardiac function under terminal urethane anesthesia (Figure 3, Table 1). Optogenetic stimulation of the DVMN prevented MI-induced reduction in LV dP/dT_max_ (7,673 ± 332 mm Hg/s in post-MI rats expressing ChiEFtdTomato vs. 5,656 ± 495 mmHg/s in post-MI rats expressing eGFP; p < 0.001), in LVESP (122 ± 4 mm Hg in post-MI rats expressing ChiEFtdTomato vs. 106 ± 3 mm Hg in post-MI rats expressing eGFP; p = 0.019), and in mean arterial pressure (78 ± 3 mm Hg in post-MI rats expressing ChiEFtdTomato vs. 66 ± 3 mm Hg in post-MI rats expressing eGFP; p = 0.043) (Figure 3). An increase in LVEDP associated with the development of LV dysfunction was reduced by vagal efferent stimulation (5.0 ± 0.7 mm Hg in post-MI rats expressing ChiEFtdTomato vs. 7.5 ± 0.6 mm Hg in post-MI rats expressing eGFP; p = 0.024) (Figure 3). Significant increases in LV dP/dT_max_ and LVESP as a result of optogenetic stimulation of the DVMN neurons were also observed in sham-operated animals (Figure 3). There were no differences in heart rate (measured under urethane anesthesia) between the experimental groups (Table 1).

## Discussion

The data obtained in the present study showed that optogenetic stimulation of vagal pre-ganglionic neurons of the DVMN (optoVNS) for 15 min every 48 h over 4 weeks following LAD occlusion preserved LV systolic function and maintained exercise capacity in a rat model of heart failure. These data are consistent with the results of earlier studies that showed that optogenetic stimulation of the DVMN neuronal activity protects the heart against acute ischemia/reperfusion injury in a rat model of MI (11), and also increases LV contractile responses to β-adrenoceptor stimulation and improves exercise capacity in healthy rats (24). Together, these data suggested that stimulation of vagal efferent activity originating from the DVMN might be critically important to confer the therapeutic benefit of VNS in heart failure.

Cardiac vagal efferent activity originates from populations of vagal pre-ganglionic neurons residing in 2 brainstem nuclei: the DVMN and the nucleus ambiguus (37,38). Control of pacemaker tissue, and thus, heart rate is provided predominantly by neurons of the nucleus ambiguus. Although most of the DVMN neurons innervate the visceral organs, a subpopulation of these cells with cardiac projections provides functional innervation of the LV and modulates its electrical (39) and contractile properties (27). Activity of DVMN neurons has a relatively small effect on heart rate (37).

The intermittent stimulation of the DVMN (which lacks major chronotropic effects) preserved cardiac function in rats with MI (as shown in this study), which was consistent with the results of some previous reports that suggested that the beneficial effects of VNS in heart failure were not entirely dependent on lowering heart rate (13, 14, 15). This was an important conclusion to draw in the context of a strong clinical association between low chronotropic vagal tone and the risk of death after MI or in established heart failure (40,41), and of the effectiveness of pharmacological interventions that lower heart rate (e.g., β-blockers and I_f_ inhibitors) (42, 43, 44). In most clinical trials of VNS in heart failure, the stimulating electrodes were placed on the right cervical vagus nerve, with the aim of lowering the heart rate, but it was not reliably attained (20).

Several mechanisms that might underlie the beneficial effects of VNS on the failing heart were proposed, including improvements of autonomic balance via sympathetic inhibition, inhibition of renin-angiotensin system activity, and anti-inflammatory effects of vagus stimulation (5,45). Selective efferent VNS applied in this study was likely to activate some or all of these mechanisms, whereas the observed effects of optogenetic stimulation of the DVMN on myocardial function in sham-operated animals suggested a plausible additional and/or alternative mechanism. Surprisingly, the effect of long-term VNS on a healthy heart has never been described, because the design of all of the preceding studies that investigated the effect of VNS in heart failure (12, 13, 14, 15,17) excluded the experimental group(s) of healthy subjects (“sham heart failure” groups) receiving the stimulation. In this study, we observed proportionally similar improvements in key measures of LV systolic function and exercise capacity in groups of rats with permanent LAD occlusion and in sham-operated animals. These data were consistent with the previously reported results that showed that optogenetic stimulation of DVMN neuronal projections enhanced myocardial contractility and responsiveness of the ventricular myocardium to β-adrenoceptor stimulation (24). These effects of DVMN stimulation were associated with reduced myocardial expression of G-protein−coupled receptor kinase 2 (GRK2) and β-arrestin 2 (24). GRKs phosphorylate β-adrenoceptors and recruit arrestins to block receptor coupling to G-proteins, which leads to receptor desensitization and internalization (46). Increased expression and activity of GRKs contribute to the progressive decline of myocardial contractile function in heart failure (47). Therefore, inhibition of GRKs was proposed as a potential therapeutic strategy of heart failure treatment (47). We recently proposed (38) that GRK2 and arrestin expression in ventricular cardiomyocytes is under parasympathetic control, with vagal withdrawal (e.g., in heart failure) leading to upregulation and enhanced vagal activity (e.g., in exercise training) leading to down-regulation of expression, with apposite changes in contractility.

Cardiac projections of a subset of the DVMN neurons innervate the LV and modulate its electrical (39) and contractile properties (27). It is plausible that optogenetic stimulation of the DVMN activates the neuronal pathways and ventricular molecular mechanisms similar to that recruited by cardiac contractility modulation, which is a novel therapeutic approach of heart failure treatment that involves application of an electrical current during the ventricular absolute refractory period (48,49). The high voltage (∼7.5V) applied in cardiac contractility modulation would be sufficient to capture the autonomic nerves, including parasympathetic fibers innervating the ventricular myocardium (50).

If this hypothesis is correct, then the VNS-based heart failure therapy should aim at stimulating the activity of vagal efferent fibers innervating the ventricles. VNS devices used in clinical trials conducted to date were not developed with an attempt to recruit specific vagal projections. Electrical stimulation applied to the cervical vagus preferentially activates sensory fibers because they have a lower activation threshold than efferent fibers (51). Experiments in large animals (pigs and dogs) demonstrated that low current stimulation (up to ∼2 mA) recruits afferent fibers predominantly and activates autonomic reflex pathways, which lead to increases in heart rate due to inhibition of vagal activity centrally (52,53). Application of higher VNS currents (>2.5 mA) is required for stimulation of efferent fibers to achieve vagally mediated lowering of the heart rate (53). No changes in heart rate in response to electrical VNS are observed when the effects of afferent and efferent fiber stimulation are in balance. These studies led to the development of a concept of a “neural fulcrum,” which was defined as the operating point based on the frequency–amplitude-pulse width of VNS, in which the stimulation has no effect on heart rate (51). The efficacy of VNS applied within the neural fulcrum is currently being tested in the ongoing ANTHEM-HFrEF trial (54). Because recruitment of efferent fibers requires more aggressive stimulation, simultaneous capture of vagal afferents may lead to significant side effects, including dysphonia, neck pain, and cough, as reported by the investigators of the NECTAR-HF trial, in which no beneficial effect of VNS on LV function was observed (20).

## Conclusions

The data obtained in this study suggest that stimulation of vagal efferent innervation of the ventricles might be critically important to deliver the therapeutic benefit of VNS in chronic heart failure.

A revised approach to the electrical VNS may be necessary to minimize the side effects that currently restrain the intensity of stimulation required for the effective recruitment of cardiac vagal efferent activity. The data reported here might be important for further development of the electrical VNS technology for targeted stimulation of a select group or subset of fibers within the trunk of the vagus nerve. This would also require detailed functional anatomical mapping of the fascicular organization of the human cervical vagus (22); this work is ongoing. Although optogenetic techniques are currently limited to preclinical research, advances in human gene therapy point the way toward eventual clinical applications of the technology. VNS using light (optoVNS) can be applied at the cervical level, and thus, can circumnavigate limitations posed by the complex anatomy of the vagus nerve and nonspecific nature of the whole nerve electrical stimulation.

Perspectives **COMPETENCY IN MEDICAL KNOWLEDGE:** VNS has been shown to slow the progression of myocardial remodeling and dysfunction in animal models of chronic heart failure. However, several multicenter clinical trials designed to test the efficacy of VNS in large cohorts of patients with heart failure did not demonstrate similar benefits with respect to the primary clinical endpoints. The nonselective nature of VNS, delivered by implantable stimulators placed on the nerve at the cervical level, may account for the failure to translate promising results of preclinical studies. To maximize the efficacy and limit the side effects, further development of VNS as a potential treatment for heart failure may require methods that allow selective stimulation of specific groups or subsets of vagal fibers.

**TRANSLATIONAL OUTLOOK:** Stimulation of vagal efferent projections to the ventricle may be critically important to deliver the therapeutic benefit of VNS in heart failure. A refined approach to VNS may be necessary to minimize the side effects that currently restrain the intensity of stimulation, which is required for the recruitment of cardiac vagal efferent innervation. Although optogenetic techniques are currently limited to research in animals, advances in human gene therapy point the way toward eventual clinical applications of the technology. VNS using light (optoVNS) can be delivered at the cervical level, and therefore, can circumnavigate limitations posed by the complex anatomy of the cervical vagus nerve and the nonspecific nature of the whole nerve electrical stimulation.
